# Interventional Radiology Procedures and Anesthesia Practices: A Bibliometric Analysis

**DOI:** 10.7759/cureus.83324

**Published:** 2025-05-01

**Authors:** Moosa Alwardi, Amber Papalkar, Abhijit Nair

**Affiliations:** 1 Radiology, Ibra Hospital, Ibra, OMN; 2 Radiology, Krishna Institute of Medical Sciences, Hyderabad, IND; 3 Anesthesiology, Ibra Hospital, Ibra, OMN

**Keywords:** `anesthesia, bibliometric analyis, interventional studies, radiology, vos-viewer

## Abstract

In order to ensure patient safety, comfort, and ideal procedural conditions, anesthesia services are essential to current interventional radiology (IR) procedures. Clinical needs, procedure type, and patient age impact the anesthetic strategy. Both adult and pediatric IR interventions are considerably more successful when the anesthesia plan is well-coordinated. In recent years, many articles in peer-reviewed journals have been published highlighting various IR procedures performed under different anesthetic approaches, such as monitored anesthesia care with sedation, general anesthesia, and regional anesthesia, successfully. Several review articles have also discussed the anesthetic facilities and monitoring required in the IR suite. This bibliometric analysis aimed to provide comprehensive details of the journals, citations, authors, and institutions that published articles on anesthesia management for IR procedures.

## Introduction and background

In recent years, interventional radiology (IR) procedures have grown dramatically, offering less invasive alternatives to conventional surgery. These treatments include pain relief procedures, tumor ablations, and vascular interventions. Anesthesia services are essential to maintaining patient safety and procedural success due to the complexity of these procedures and the need for patient comfort and immobility [1-5]. Several IR procedures, including vascular access and biopsy, can be carried out under local anesthesia (LA) [6]. For procedures that require sedation while preserving spontaneous breathing, monitored anesthesia care (MAC) is commonly employed. In certain situations, such as limb interventions, regional anesthesia (RA) is used. For complex procedures like transjugular intrahepatic portosystemic shunt (TIPS) placement, embolization of large tumors, or when patients are uncooperative, general anesthesia (GA) is preferred [7].

Anesthesiologists enhance patient safety and comfort by providing sedation, alleviating anxiety, facilitating longer and more complex procedures without patient movement, and allowing IR specialists to focus entirely on technical aspects without managing sedation-related issues. The success of any IR intervention depends on close coordination between anesthesiologists, radiologists, and nursing staff, and effective communication further enhances procedural efficiency and reduces complications [8,9]. Several IR procedures involve vascular manipulation, necessitating close monitoring of blood pressure and cardiac function [10-12]. GA cases may require advanced airway management, particularly in prone or lateral positions. Sedation strategies must be optimized to prevent patient discomfort while ensuring cooperation [13,14]. RA has also been successfully utilized in many IR procedures [15]. Most pediatric patients require deep sedation or GA due to their inability to remain still. Neonates and infants have unique respiratory and cardiovascular considerations, requiring tailored anesthetic management [16-19]. Pediatric vascular access is typically challenging and time-consuming [20,21]. To deliver safe and effective anesthetic care, the IR suite must be equipped with a fully functional anesthesia workstation. Patient safety during procedures is ensured by standard monitoring, which includes ECG, pulse oximetry, capnography, and invasive blood pressure monitoring when necessary. To manage anesthesia-related emergencies such as hemodynamic instability, anaphylaxis, or airway compromise, a fully stocked crash cart must be readily accessible. Immediate access to defibrillators, emergency medications, and advanced airway management tools is essential for timely intervention in the event of complications [22,23].

The type of quantitative research used to evaluate academic literature is bibliometric analysis, which examines networks, patterns, and trends within a specific area of study. This method utilizes tools and validated software to assess research productivity, citation dynamics, and collaborations among researchers, institutions, and countries. Bibliometric studies provide valuable insights into the evolution of knowledge, highlight research gaps, and identify influential works, all of which help guide further research [24-26]. This bibliometric analysis aims to systematically analyze the trends and hotspots of research involving IR and anesthesia practices over the past five years.

## Review

Methods

Search Strategy

We searched the literature from January 2020 to March 2025. The literature search and data downloads were performed on a single day (24th March 2025) to minimize bias arising from database updates. A comprehensive search was conducted on the Scopus database using the following search strategy: TITLE-ABS-KEY (interventional AND radiology AND anesthesia) AND PUBYEAR > 2019 AND PUBYEAR < 2026 AND (EXCLUDE (DOCTYPE, "tb") OR EXCLUDE (DOCTYPE, "er")) AND (EXCLUDE (SUBJAREA, "VETE") OR EXCLUDE (SUBJAREA, "ENVI") OR EXCLUDE (SUBJAREA, "ENGI") OR EXCLUDE (SUBJAREA, "PHYS") OR EXCLUDE (SUBJAREA, "CENG")) AND (EXCLUDE (LANGUAGE, "French") OR EXCLUDE (LANGUAGE, "Chinese") OR EXCLUDE (LANGUAGE, "Spanish") OR EXCLUDE (LANGUAGE, "German") OR EXCLUDE (LANGUAGE, "Russian") OR EXCLUDE (LANGUAGE, "Japanese"))

The Scopus file was stored in comma-separated values (CSV) format. The CSV file was systematically screened by two authors (MAW and AN). Articles that did not involve anesthesia management were removed. The final set of articles was processed for bibliometric analysis.

Data Processing

Complete records, after removing ineligible articles and cited references, were included in the dataset, which was then exported for further analysis. For bibliometric analysis, VOSviewer (Version 1.6.20, Leiden University, Netherlands) was used. We planned five types of analysis using VOSviewer [27]: bibliographic coupling, citation analysis, co-authorship analysis, co-occurrence analysis, and co-citation analysis. The units of analysis for co-authorship analysis were countries, organizations, and authors. For co-occurrence analysis, the units were index keywords, author keywords, and all keywords. Citation analysis and bibliographic coupling were performed using documents, sources, authors, organizations, and countries as units of analysis. For co-citation analysis, the units of analysis were cited authors, cited sources, and cited references.

Results

A Scopus database search revealed 633 documents. After screening all documents and excluding those that did not include both IR and anesthesia, 267 documents fulfilled the inclusion criteria. The CSV file was then uploaded to the VOSviewer software for bibliometric analysis across various categories.

Co-authorship Analysis

Co-authorship and authors: Out of 1,627 authors, only 6 met the threshold. Bairbre L. Connolly had 3 documents with 55 citations, and Andreas H. Mahnken had 3 documents with 32 citations (Table 1).

**Table 1:** Co-authorship and authors.

Author	Documents	Citations
Connolly, Bairbre L.	3	55
Han, Xinwei	3	21
Jiao, Dechao	3	18
Mahnken, Andreas H.	3	32
Prasad, Surya Nandan	5	11
Singh, Vivek	5	11

Co-authorship and organizations: Out of 842 organizations, 6 met the threshold. PGIMS in Lucknow, India, had 3 documents, while the remaining 5 organizations had 2 documents each. However, the Vascular and Interventional Radiology Department at Careggi University Hospital, Florence, Italy, had a total of 42 citations, the highest among all (Table 2).

**Table 2:** Co-authorship and organizations.

Organization	Documents	Citations
Department of Biotechnological and Applied Clinical Sciences, University of L’Aquila, L’Aquila, Italy	2	15
Department of Interventional Radiology, Duke University Medical Center, Durham, United States	2	5
Department of Interventional Radiology, The First Affiliated Hospital of Zhengzhou University, China	2	3
Department of Radiology, Mayo Clinic, Rochester, United States	2	12
Department of Radiodiagnosis, Sanjay Gandhi Postgraduate Institute of Medical Sciences, Uttar Pradesh, India	3	11
Vascular and Interventional Radiology Department, Careggi University Hospital, Florence, Italy	2	42

**Figure 1:**
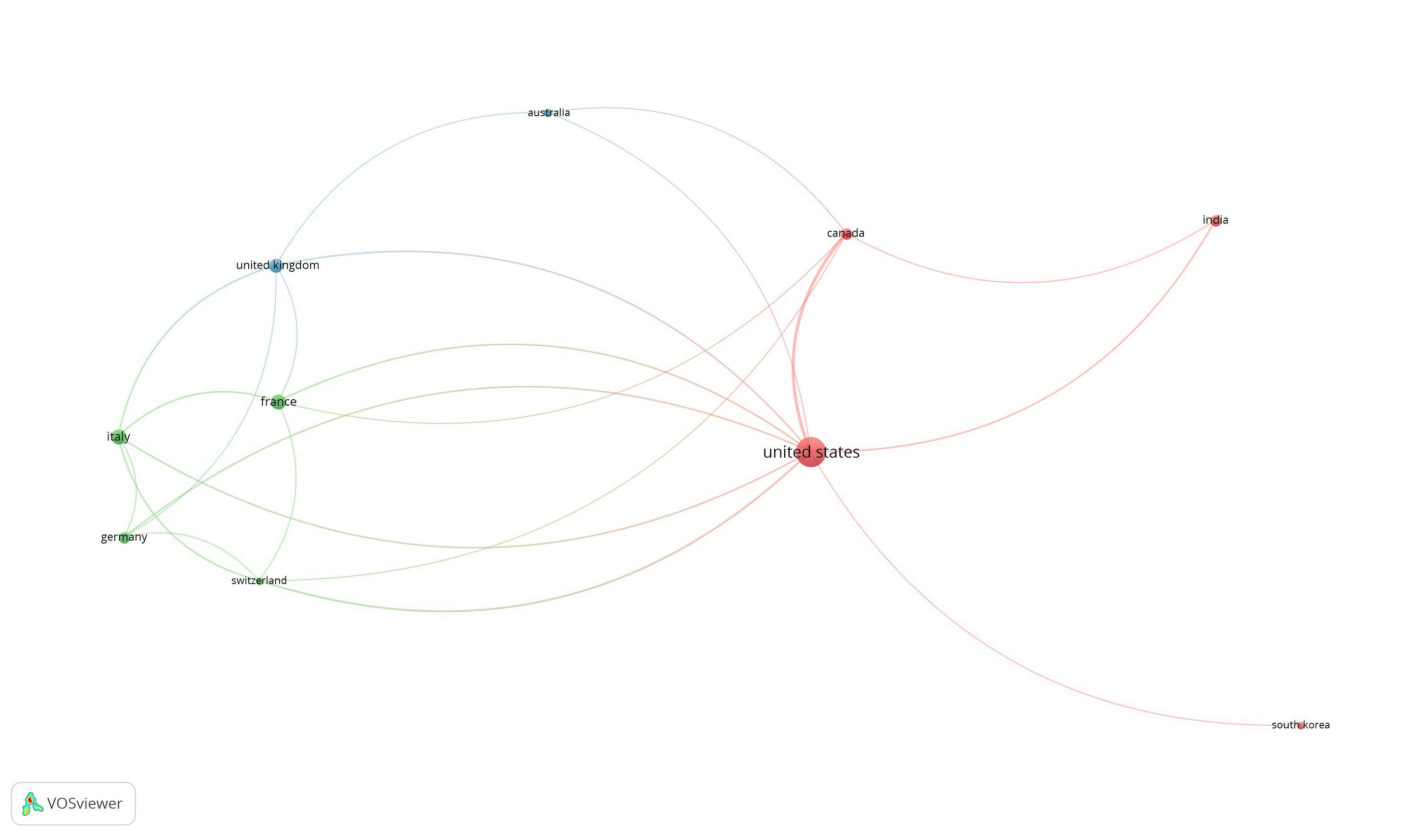
Mapping of co-authorship and countries.

Co-authorship and countries: Out of 54 countries, 14 met the threshold. The top 10 countries are summarized in Figure 1. The United States had the highest output, with 87 documents and 335 citations (Table 3).

**Table 3:** Co-authorship and countries.

Country	Documents	Citations
Australia	7	46
Canada	14	125
France	24	95
Germany	12	50
India	14	24
Italy	24	118
South Korea	6	14
Switzerland	6	48
United Kingdom	19	65
United States	87	335

Co-occurrence Analysis

**Figure 2:**
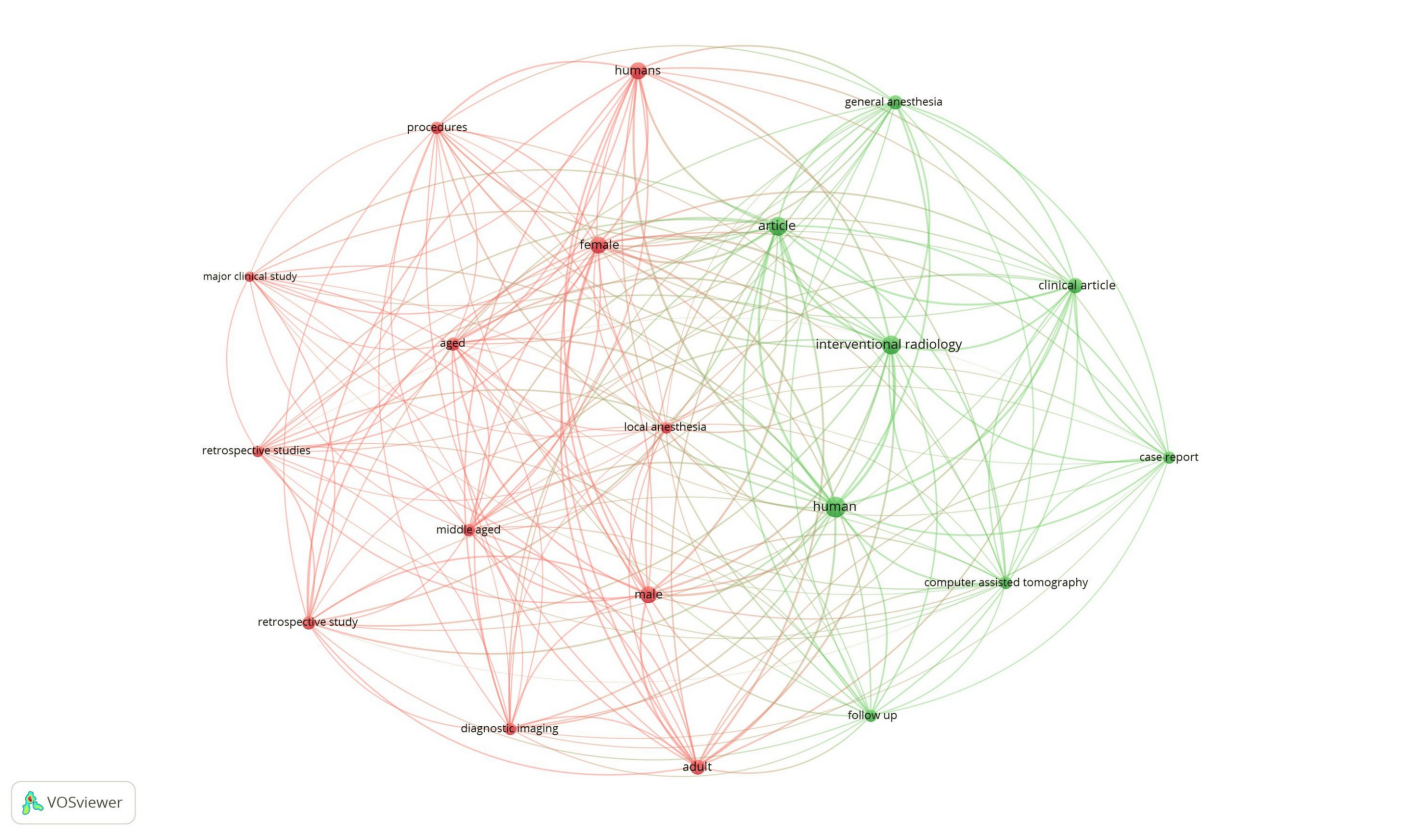
Mapping of co-occurrence and all keywords.

Co-occurrence and all keywords: Of the 4,102 keywords, 487 met the threshold. A network was generated for the 20 most frequently occurring keywords. The resulting network diagram is shown in Figure 2.

**Figure 3:**
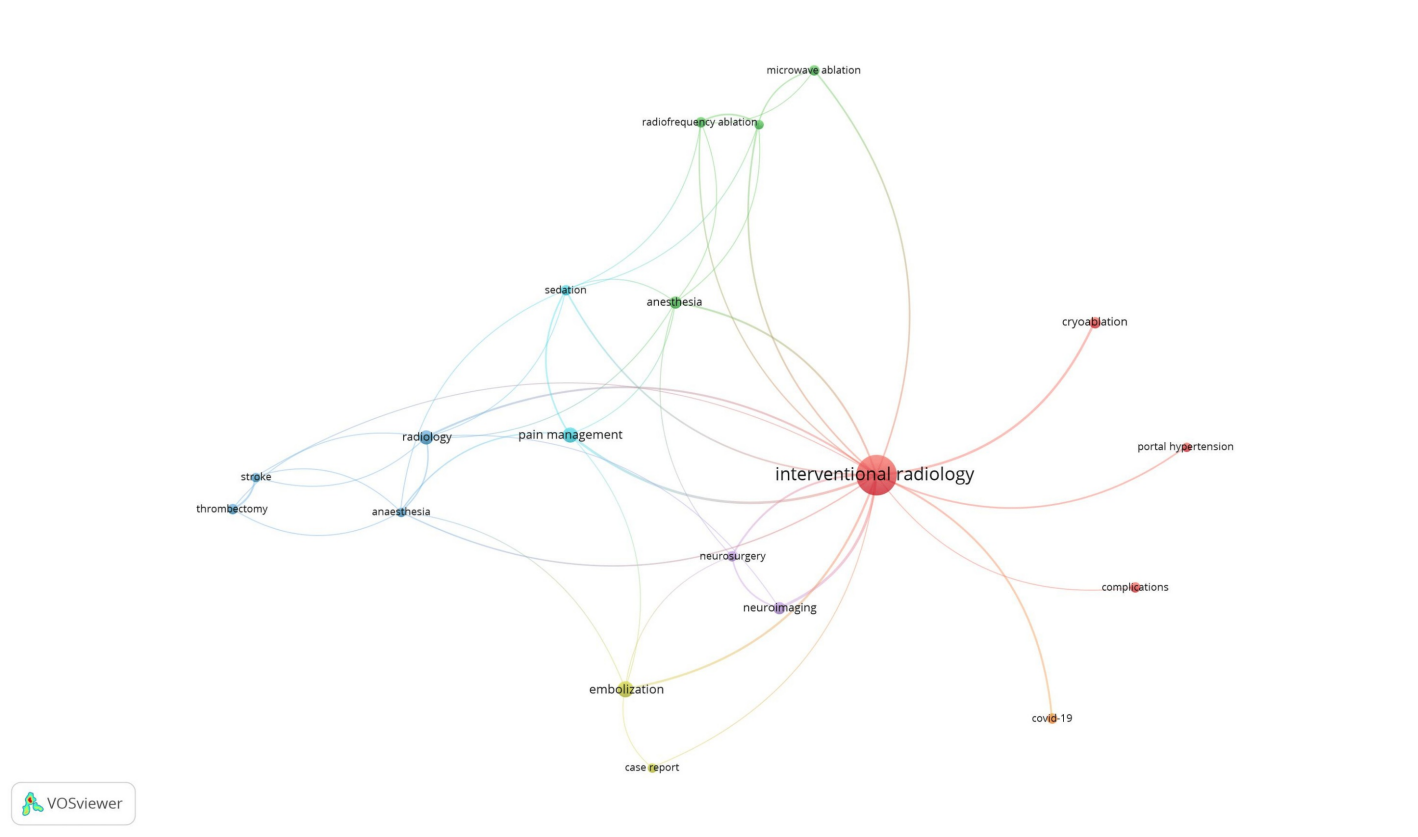
Mapping of co-occurrence and author keywords.

Co-occurrence and author keywords: Out of 670 keywords, 19 met the threshold, all of which were used to create the network. Interventional Radiology was the most common author keyword, with 79 occurrences and a total link strength of 61. The network diagram is shown in Figure 3.

**Figure 4:**
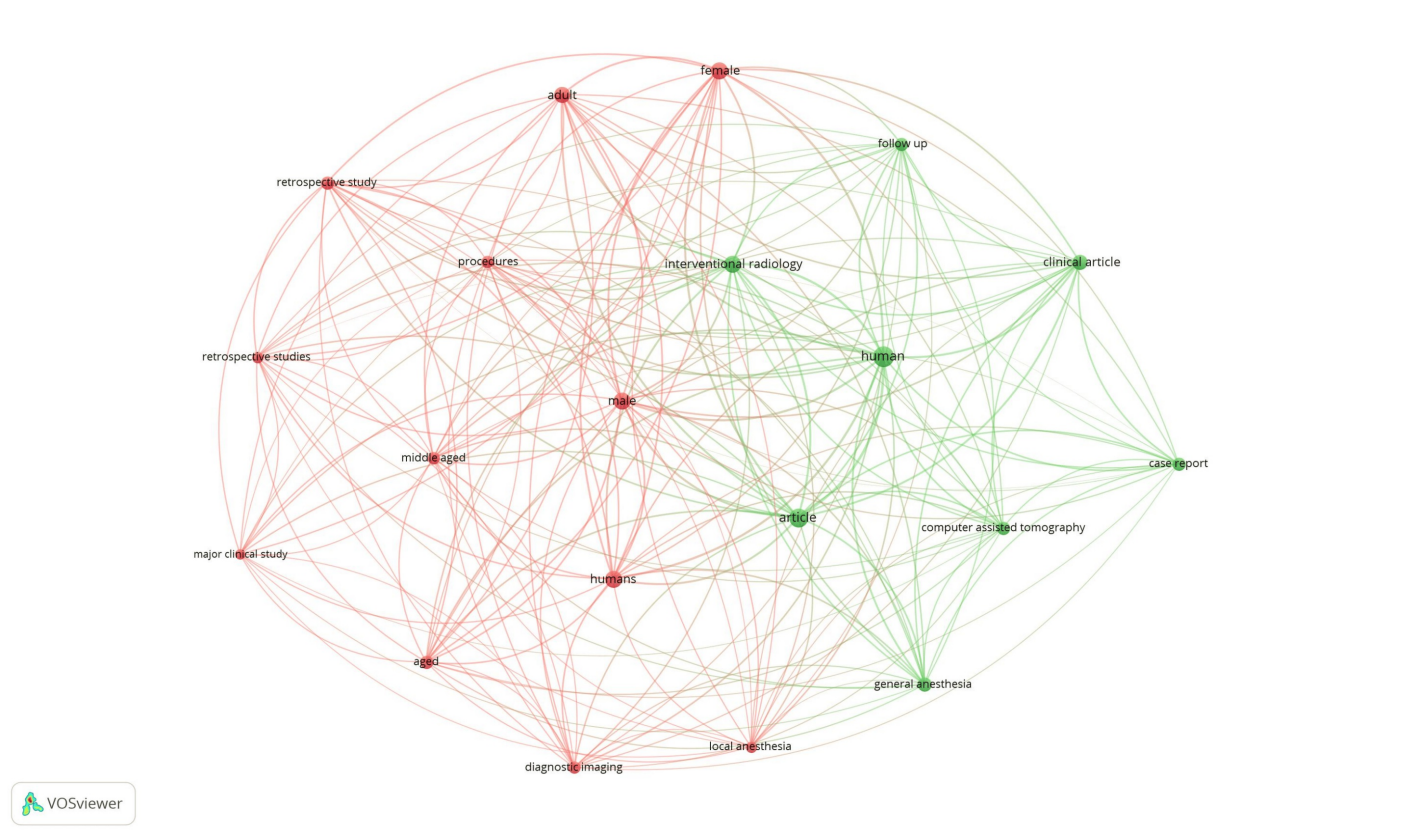
Mapping of co-occurrence and index keywords.

Co-occurrence and index keywords: Of the 3,705 keywords, 463 met the threshold. We selected the top 20 keywords to create the network. The network diagram is shown in Figure 4.

Citation Analysis

Citation analysis and documents: Out of the 267 documents, 112 met the threshold. We selected 20 documents to create the network, of which only 3 were connected. Romagnoli (2020) had 25 citations, the highest in this category [28].

**Figure 5:**
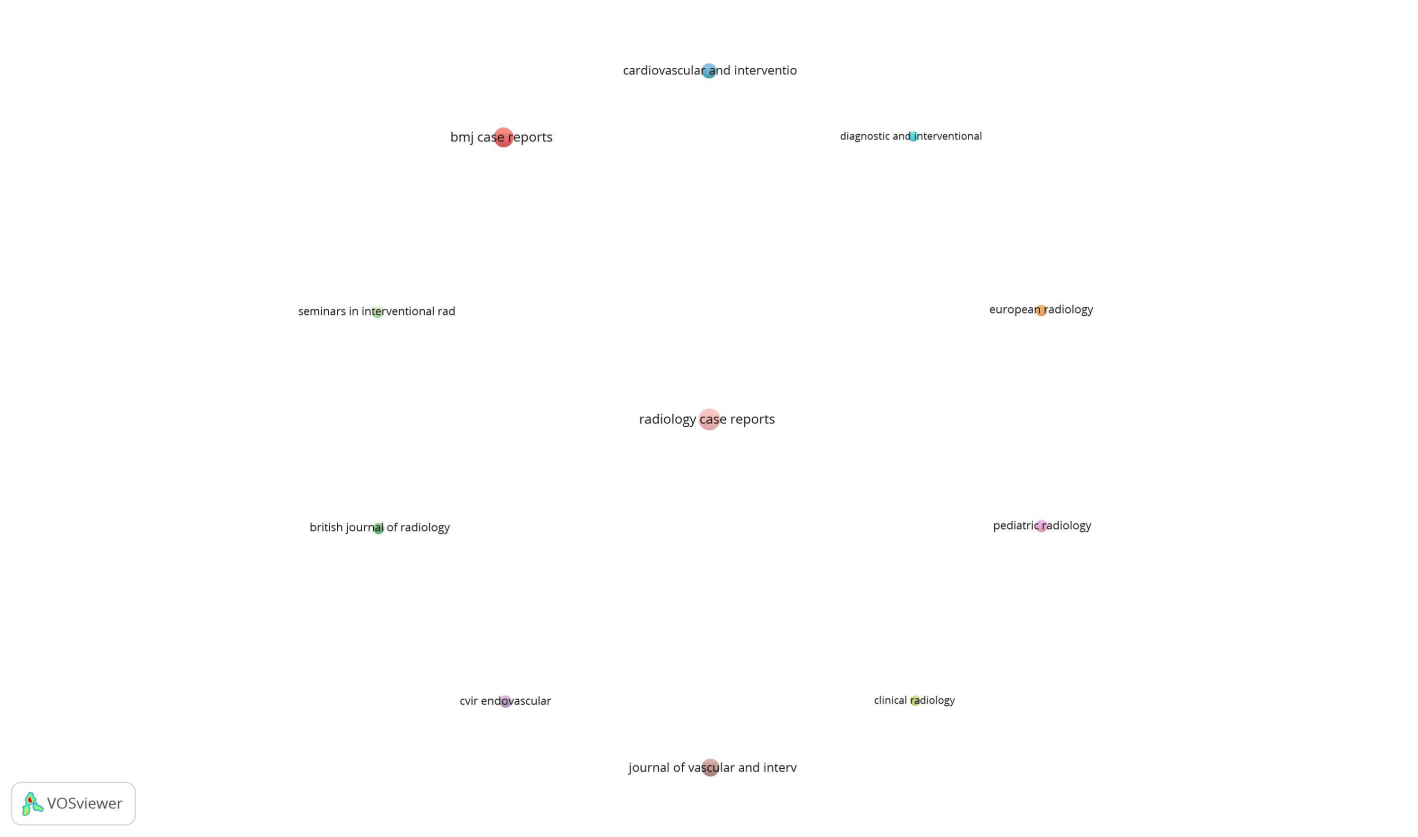
Mapping of citation analysis and sources.

Citation analysis and sources: Of the 137 sources, 11 met the threshold. All 11 sources were included to create the network (Figure 5). Radiology Case Reports had the highest number of documents (21), with 22 citations. However, Journal of Vascular and Interventional Radiology had the highest number of citations (150), based on 14 documents (Table 4).

**Table 4:** Citation analysis and sources.

Source	Documents	Citations
BMJ Case Reports	17	30
British Journal of Radiology	6	33
Cardiovascular and Interventional Radiology	10	41
Clinical Radiology	5	10
CVIR Endovascular	7	11
Diagnostic and Interventional Imaging	5	44
European Radiology	6	36
Journal of Vascular and Interventional Radiology	14	150
Pediatric Radiology	7	13
Radiology Case Reports	21	22
Seminars in Interventional Radiology	6	7

Citation analysis and authors: Of the 1,558 authors, 6 met the threshold. A network was created using all six authors. Surya Nandan Prasad and Vivek Singh each had five documents with 11 citations (Table 5).

**Table 5:** Citation analysis and authors.

Author	Documents	Citations
Connolly, Bairbre l.	3	55
Han, Xinwei	3	21
Jiao, Dechao	3	18
Mahnken, Andreas H.	3	32
Prasad, Surya Nandan	5	11
Singh, Vivek	5	11

Citation analysis and organizations: Of the 842 organizations, 6 met the threshold. The details are summarized in Table 6.

**Table 6:** Citation analysis and organizations.

Organization	Documents	Citations
Department of Biotechnological and Applied Clinical Sciences, University of L’Aquila, L’Aquila, Italy	2	15
Department of Interventional Radiology, Duke University Medical Center, Durham, United States	2	5
Department of Interventional Radiology, The First Affiliated Hospital of Zhengzhou University, China	2	3
Department of Radiology, Mayo Clinic, Rochester, United States	2	12
Department of Radiodiagnosis, Sanjay Gandhi Postgraduate Institute of Medical Sciences, Uttar Pradesh, India	3	11
Vascular and Interventional Radiology Department, Careggi University Hospital, Florence, Italy	2	42

**Figure 6:**
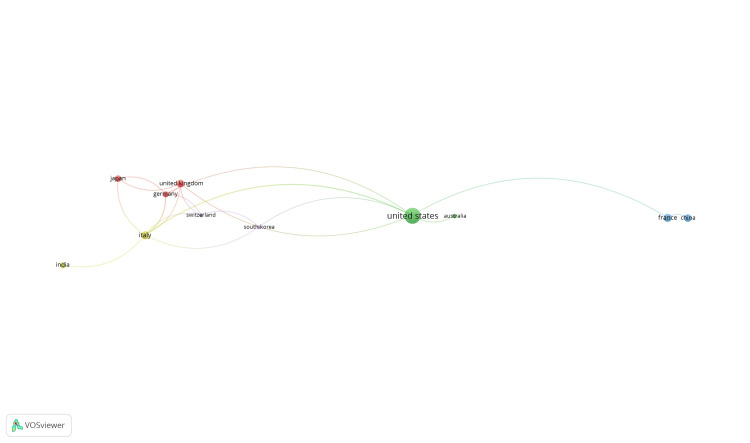
Mapping of citation analysis and countries.

Citation analysis and countries: Of the 53 countries, 14 met the threshold. A network was created using all 14 countries, of which 11 were connected (Figure 6). The United Kingdom had the highest number of documents (87) and 335 citations.

Bibliographic Coupling

**Figure 7:**
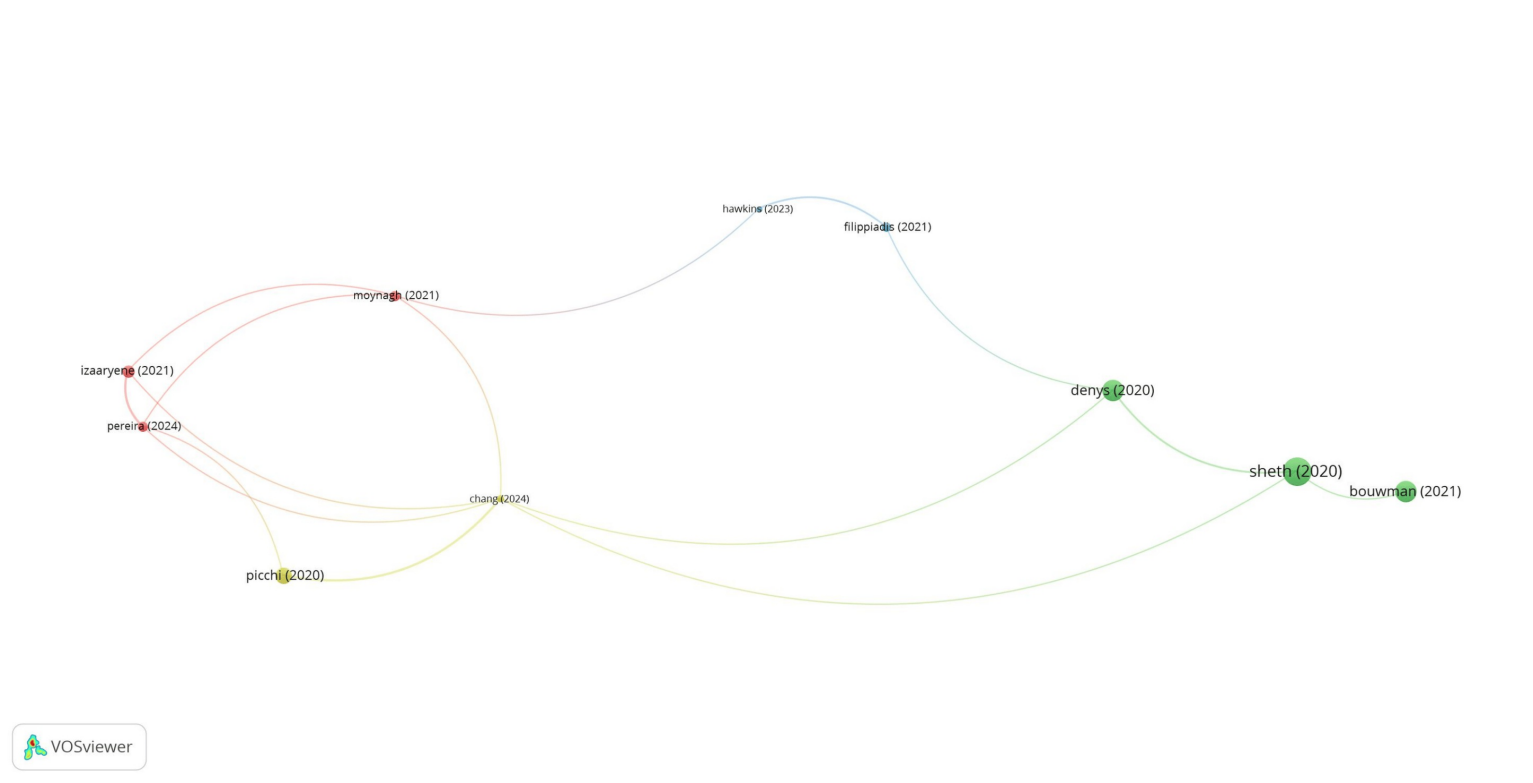
Mapping of bibliographic coupling and documents.

Bibliographic coupling and documents: Of the 267 documents, 112 met the threshold. A network of 20 documents was created, of which 10 were connected (Figure 7). Bouwman had 31 citations, followed by Denys, who had 30 citations.

**Figure 8:**
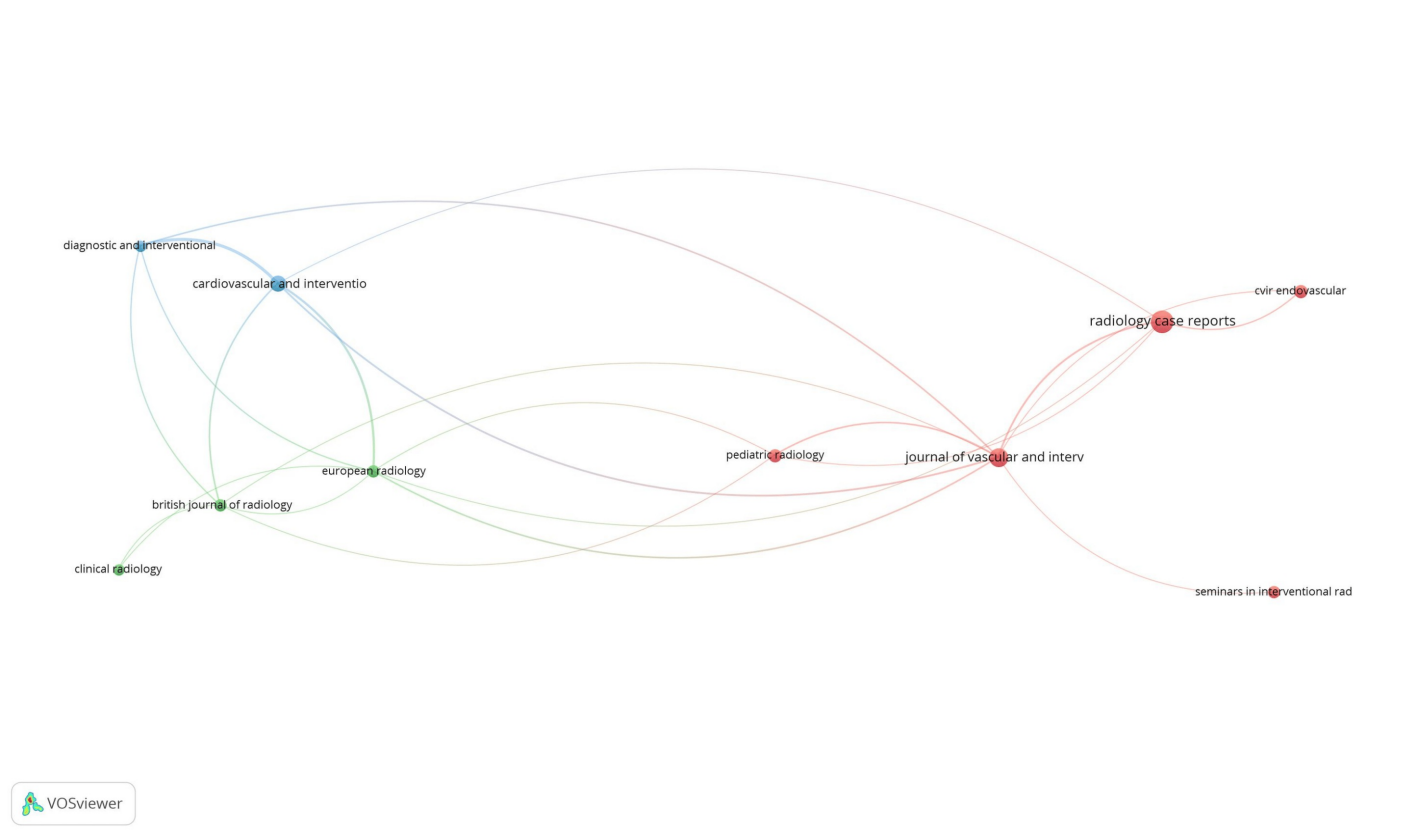
Mapping of bibliographic coupling and sources.

Bibliographic coupling and sources: Of the 137 sources, 11 met the threshold, with 10 connected and used to create the network (Figure 8). Journal of Vascular and Interventional Radiology had 150 citations from 5 sources.

Bibliographic coupling and authors: Of the 1,558 authors, 6 met the threshold. Author Bairbre L. Connolly had 55 citations from 3 documents.

Bibliographic coupling and organizations: Of the 847 organizations, 7 met the threshold. The Vascular and Interventional Radiology Department at Careggi University Hospital, Florence, Italy, had 42 citations from its two documents.

**Figure 9:**
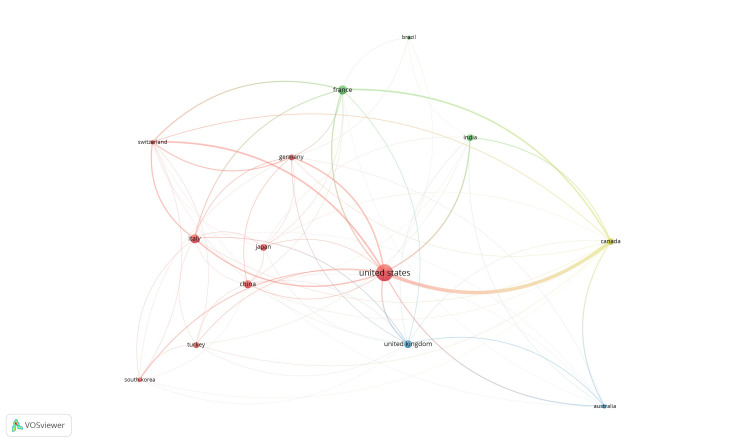
Mapping of bibliographic coupling and countries.

Bibliographic coupling and countries: Of the 54 countries, 14 met the threshold and were included in the network (Figure 9). With 335 citations from 35 documents, the USA was the leading country.

Co-citation Analysis

Co-citation and cited references: Out of 6,418 references, 6 met the threshold and were used to create the network. The article by MacMahon et al. had the highest number of citations (4) (Table 7).

**Table 7:** Co-citation and cited references.

cited reference	citations
Dindo D et al. (2004) [29]	3
Gillams A et al. (2015) [30]	3
Khalilzadeh O et al. (2017) [31]	3
MacMahon H et al. (2017) [32]	4
American Society of Anesthesiologists Task Force on Sedation and Analgesia by Non-Anesthesiologists (2002) [33]	3
Santiago E et al. (2018) [34]	3

**Figure 10:**
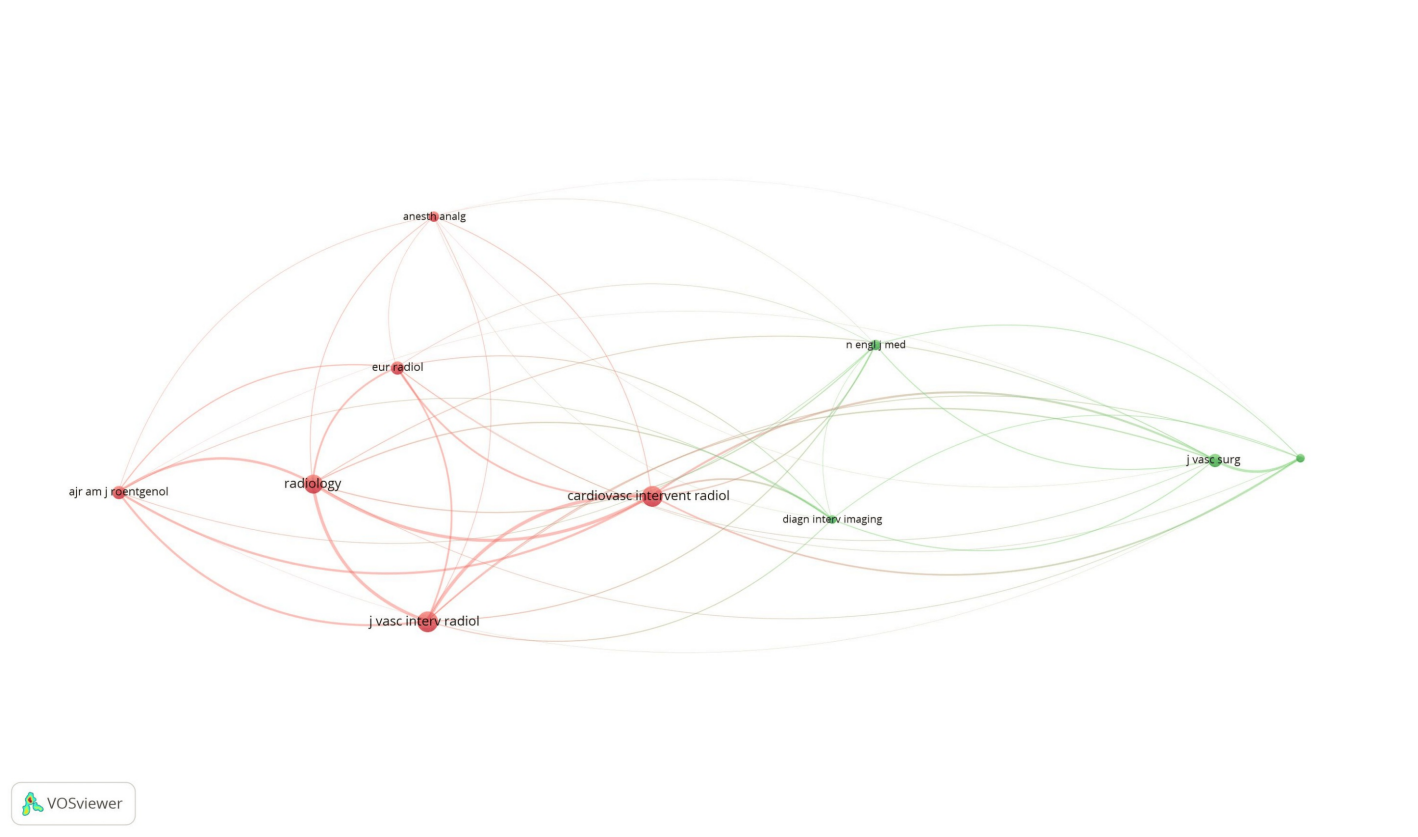
Mapping of co-citation and cited sources.

Co-citation and cited sources: Of the 2,180 sources, 38 met the threshold. We selected the top 10 sources to create the network (Figure 10). The Journal Cardiovascular and Interventional Radiology had 206 citations, followed by Journal of Vascular and Interventional Radiology with 203 citations (Table 8).

**Table 8:** Co-citation and cited sources.

Source	Citations
AJR Am J Roentgenol	83
Anesth Analg	52
Cardiovasc Intervent Radiol	206
Diagn Interv Imaging	39
Eur J Vasc Endovasc Surg	37
Eur Radiol	86
J Vasc Interv Radiol	203
J Vasc Surg	84
N Engl J Med	51
Radiology	175

Co-citation and cited authors: Of the 18,345 authors, 3 met the threshold. Cazzato RL had the highest number of citations in this category, with a total of 29.

Discussion

This bibliometric analysis has attempted to identify emerging trends and significant research related to IR and anesthesia practices.

Recent years have seen tremendous advancements in IR, enhancing patient outcomes through the incorporation of artificial intelligence (AI), improved procedural techniques, and advanced imaging technologies. These innovations have transformed IR into a field capable of providing highly precise, minimally invasive treatments for a wide spectrum of diseases [35]. Advancements in catheter design and related technologies have significantly improved the safety and efficacy of interventional procedures. With increased stability and flexibility, modern catheters facilitate navigation through complex anatomy. By reducing systemic side effects, improving therapeutic efficacy, and enabling the precise delivery of targeted medications, developments in IR have revolutionized vascular interventions [36]. Transcatheter arterial chemoembolization, which combines chemotherapy and embolization, has proven effective in treating liver tumors. The integration of AI into IR holds the potential to substantially enhance procedural efficiency and diagnostic accuracy. AI algorithms can analyze imaging data to assist radiologists in detecting anomalies and determining optimal treatment strategies. Predictive analytics can facilitate personalized treatment planning by evaluating large datasets to anticipate outcomes and potential side effects. AI-driven solutions also support workflow optimization through real-time data analysis and automated scheduling systems, thereby increasing patient flow and operational efficiency [37].

Robot-assisted interventions have improved control and precision during IR procedures, enhancing outcomes and shortening recovery periods [38]. Augmented reality and three-dimensional imaging provide more comprehensive views of pathology, enabling even greater procedural accuracy [39]. These technological advances have contributed to significantly improved patient outcomes. Modern IR procedures are minimally invasive, which reduces the risk of complications, shortens recovery time, and improves overall patient satisfaction.

Recent developments in procedural sedation and analgesia for non-operating room anesthesia (NORA) have focused on improving patient outcomes, safety, and efficiency [40,41]. Advances in drug delivery systems now allow for greater precision in administration, enabling tailored sedation that meets individual patient needs. These systems reduce the risk of both under- and oversedation, thereby improving safety. Technologies such as target-controlled infusion (TCI) pumps, Bispectral Index (BIS) monitoring, and capnography for conscious sedation are now prioritized in IR suites by various societies and institutions [42-44].

Anesthetists providing services in IR settings must be well-versed in the pharmacokinetics and pharmacodynamics of anesthetic agents. They must also be prepared to manage complications such as overdose or respiratory events due to airway obstruction. Patients undergoing IR are often critically ill, with low cardiopulmonary reserves. When indicated, based on comorbidities and procedural complexity, thorough pre-procedure evaluations are essential [45-47].

Due to its opioid-sparing properties, RA has become increasingly popular in pediatric IR. A retrospective study conducted from January 2016 to September 2022 investigated the effects of nerve blocks during IR-performed sclerotherapy for vascular malformations and bone cysts [48]. The study found that patients who received nerve blocks required significantly less opioid medication during and after procedures. As IR procedures become increasingly complex, the need for specialized anesthetic support continues to grow. Anesthesiologists must work closely with radiologists to ensure patient safety and must be proficient in the specific requirements of IR procedures [49,50].

There are several limitations to this bibliometric analysis. We only searched the Scopus database, although other databases suitable for bibliometric analysis include Web of Science, PubMed, Lens, and Dimensions. However, the software used allows analysis of results generated from only one database. Therefore, some relevant articles may have been missed. Additionally, articles published after the date of our data collection were excluded, as new publications were continually added to the literature after this analysis was submitted for review. Several tools are available for bibliometric analysis, including CiteSpace, Bibliometrix, and Scientometrics. However, we chose to use VOSviewer because it offers advanced yet user-friendly network visualizations.

## Conclusions

This bibliometric analysis involving IR and anesthesia has attempted to analyze the current literature related to these two entities. A tailored anesthesia approach is vital to modern interventional radiology procedures, ensuring patient safety, comfort, and optimal procedural conditions. The results of this analysis may be helpful for researchers planning to conduct further studies involving collaboration between IR and anesthesia management, thereby identifying new directions for future research.

## References

[1] Naidu SG, Narayanan H, Saini G, Segaran N, Alzubaidi SJ, Patel IJ, Oklu R (2021). Prostate artery embolization-review of indications, patient selection, techniques and results. J Clin Med.

[2] Ahmed O, Block J, Mautner K (2021). Percutaneous management of osteoarthritis in the knee: proceedings from the Society of Interventional Radiology Research Consensus Panel. J Vasc Interv Radiol.

[3] Moynagh MR, Dowdy SC, Welch B (2021). Image-guided tumor ablation in gynecologic oncology: review of interventional oncology techniques and case examples highlighting a collaborative, multidisciplinary program. Gynecol Oncol.

[4] Fletcher A, Moore KJ, Stensby JD, Hulbert A, Saemi AM, Davis RM, Bhat AP (2021). The pain crisis: interventional radiology's role in pain management. AJR Am J Roentgenol.

[5] Hayek G, Kastler B (2020). Interventional radiology for treatment of bone metastases. Cancer Radiother.

[6] Li X, Trerotola SO (2022). Local anesthesia in interventional radiology. Semin Intervent Radiol.

[7] Landrigan-Ossar M (2015). Common procedures and strategies for anaesthesia in interventional radiology. Curr Opin Anaesthesiol.

[8] Lee MJ, Fanelli F, Haage P, Hausegger K, Van Lienden KP (2012). Patient safety in interventional radiology: a CIRSE IR checklist. Cardiovasc Intervent Radiol.

[9] Wijayatilake DS, Ratnayake G, Ragavan D (2016). Anaesthesia for neuroradiology: thrombectomy: 'one small step for man, one giant leap for anaesthesia'. Curr Opin Anaesthesiol.

[10] Sou BS, Aglio LS, Zhou J (2021). Anesthetic management of acute ischemic stroke in the interventional neuro-radiology suite: state of the art. Curr Opin Anaesthesiol.

[11] Bravo E, Tempesta D, Viault N (2025). Improving pre- and post-IR procedure experience: What the anesthesiologists can offer. Cardiovasc Intervent Radiol.

[12] Keefe N, Patel N, Mody P (2024). Obstetric interventional radiology: periprocedural considerations when caring for the pregnant and postpartum patient. Semin Intervent Radiol.

[13] Bello C, Paisansathan C, Riva T, Luedi MM, Andereggen L (2022). Anesthesia care in the interventional neuroradiology suite: an update. Curr Opin Anaesthesiol.

[14] Piccioni F, Poli A, Templeton LC (2019). Anesthesia for percutaneous radiofrequency tumor ablation (PRFA): a review of current practice and techniques. Local Reg Anesth.

[15] Sag AA, Qadri YJ (2022). Interventional radiology regional anesthesia approaches for intra- and postprocedural pain control. Semin Intervent Radiol.

[16] Nelson O, Bailey PD Jr (2017). Pediatric anesthesia considerations for interventional radiology. Anesthesiol Clin.

[17] Shaikh R, Munoz FG (2021). Endovascular approaches in pediatric interventional oncology. CVIR Endovasc.

[18] Alexander MD, Halbach VV, Hetts SW (2021). "And do no harm": complications in interventional neuroradiology. Handb Clin Neurol.

[19] Roebuck DJ, Stockton E, Ritchie-McLean SN, McLaren CA (2020). Interventional radiology in the airway in children. Paediatr Anaesth.

[20] Johansen M, Classen V, Muchantef K (2021). Long-term IV access in paediatrics - why, what, where, who and how. Acta Anaesthesiol Scand.

[21] Desai SB, Kukreja KU (2018). How to recognize, avoid, or get out of trouble in pediatric interventional radiology. Tech Vasc Interv Radiol.

[22] Shabanie A (2006). Conscious sedation for interventional procedures: a practical guide. Tech Vasc Interv Radiol.

[23] Johnson S (2010). Sedation and analgesia in the performance of interventional procedures. Semin Intervent Radiol.

[24] Cooper ID (2015). Bibliometrics basics. J Med Libr Assoc.

[25] Szomszor M, Adams J, Fry R, Gebert C, Pendlebury DA, Potter RW, Rogers G (2020). Interpreting bibliometric data. Front Res Metr Anal.

[26] Lazarides MK, Lazaridou IZ, Papanas N (2023). Bibliometric analysis: bridging informatics with science. Int J Low Extrem Wounds.

[27] van Eck NJ, Waltman L (2010). Software survey: VOSviewer, a computer program for bibliometric mapping. Scientometrics.

[28] Romagnoli S, Fanelli F, Barbani F (2020). CIRSE Standards of practice on analgesia and sedation for interventional radiology in adults. Cardiovasc Intervent Radiol.

[29] Dindo D, Demartines N, Clavien PA (2004). Classification of surgical complications: a new proposal with evaluation in a cohort of 6336 patients and results of a survey. Ann Surg.

[30] Gillams A, Goldberg N, Ahmed M (2015). Thermal ablation of colorectal liver metastases: a position paper by an international panel of ablation experts, The Interventional Oncology Sans Frontières meeting 2013. Eur Radiol.

[31] Khalilzadeh O, Baerlocher MO, Shyn PB (2017). Proposal of a new adverse event classification by the Society of Interventional Radiology Standards of Practice Committee. J Vasc Interv Radiol.

[32] MacMahon H, Naidich DP, Goo JM (2017). Guidelines for management of incidental pulmonary nodules detected on CT images: from the Fleischner Society 2017. Radiology.

[33] American Society of Anesthesiologists Task Force on Sedation and Analgesia by Non-Anesthesiologists (2002). Practice guidelines for sedation and analgesia by non-anesthesiologists. Anesthesiology.

[34] Santiago E, Pauly V, Brun G, Guenoun D, Champsaur P, Le Corroller T (2018). Percutaneous cryoablation for the treatment of osteoid osteoma in the adult population. Eur Radiol.

[35] De Filippo M, Brunese L, Reginelli A (2019). Advances in diagnostic and interventional radiology. Acta Biomed.

[36] Midulla M, Pescatori L, Chevallier O (2019). Future of IR: emerging techniques, looking to the future…and learning from the past. J Belg Soc Radiol.

[37] Malpani R, Petty CW, Bhatt N, Staib LH, Chapiro J (2021). Use of artificial intelligence in non-oncologic interventional radiology: current state and future directions. Dig Dis Interv.

[38] Lanza C, Carriero S, Buijs EF (2023). Robotics in interventional radiology: review of current and future applications. Technol Cancer Res Treat.

[39] Baker J, Antypas A, Aggarwal P (2024). Augmented reality in interventional radiology: transforming training paradigms. Cureus.

[40] Schenker MP, Martin R, Shyn PB, Baum RA (2009). Interventional radiology and anesthesia. Anesthesiol Clin.

[41] Cornelis FH, Monard E, Moulin MA (2019). Sedation and analgesia in interventional radiology: Where do we stand, where are we heading and why does it matter?. Diagn Interv Imaging.

[42] Khorsand S, Karamchandani K, Joshi GP (2022). Sedation-analgesia techniques for nonoperating room anesthesia: an update. Curr Opin Anaesthesiol.

[43] Baerlocher MO, Nikolic B, Silberzweig JE, Kinney TB, Kuo MD, Rose SC (2013). Society of Interventional Radiology position statement on recent change to the ASA's moderate sedation standards: capnography. J Vasc Interv Radiol.

[44] Dhansura T, Shaikh N (2017). The parturient in the interventional radiology suite: new frontier in obstetric anaesthesia. Indian J Anaesth.

[45] Kim H, Lane J, Schlichter R, Stecker MS, Taus R (2017). Use of anesthesiology services in radiology. Anesthesiol Clin.

[46] Gross WL, Cooper L, Boggs S (2017). Demands of integrated care delivery in interventional medicine and anesthesiology: interdisciplinary teamwork and strategy. Anesthesiol Clin.

[47] Pinto A, Giurazza F, Califano T, Rea G, Valente T, Niola R, Caranci F (2021). Interventional radiology in gynecology and obstetric practice: safety issues. Semin Ultrasound CT MR.

[48] Gaelen JI, Wu C, Yang A, Rajeswaran S, Lazar A, Cheon EC, Vargas AA (2024). Use of regional anesthesia within a pediatric interventional radiology suite reduced periprocedural opioid use without delaying the overall workflow: a retrospective study. Reg Anesth Pain Med.

[49] Amin A, Lane JS (2018). The future of anesthesia for interventional radiology. Curr Opin Anaesthesiol.

[50] Castioni CA, Amadori A, Bilotta F (2017). Italian COnsensus in Neuroradiological Anesthesia (ICONA). Minerva Anestesiol.

